# The Role of Homologous Recombination Deficiency (HRD) in Renal Cell Carcinoma (RCC): Biology, Biomarkers, and Therapeutic Opportunities

**DOI:** 10.3390/curroncol32120690

**Published:** 2025-12-07

**Authors:** Alberto Bongiovanni, Pierfranco Conte, Vincenza Conteduca, Matteo Landriscina, Giuseppe Di Lorenzo, Francesco Cognetti

**Affiliations:** 1 Departmental Faculty of Medicine, UniCamillus-Saint Camillus International University of Health and Medical Sciences, 00131 Rome, Italy; 2 Periplo Foundation, 26100 Cremona, Italy; 3 Unit of Medical Oncology and CREATE Center for Research and Innovation Medicine, Department of Medical and Surgical Sciences, University of Foggia, Policlinico Riuniti, 71122 Foggia, Italy; vincenza.conteduca@unifg.it (V.C.); matteo.landriscina@unifg.it (M.L.); 4 Oncology Unit, “Andrea Tortora” Hospital, ASL Salerno, 84016 Pagani, Italy; 5 Associazione O.R.A. ETS-Oncology Research Assistance, 84134 Salerno, Italy

**Keywords:** renal cell cancer, homologous recombination deficiency (HRD), RCC, review, ATR inhibitors

## Abstract

Renal Cell Carcinoma (RCC) is a common malignancy, often diagnosed incidentally. In recent years, the prognosis of metastatic disease has been improved due to the development of immune checkpoint inhibitors (ICI) and tyrosine kinase inhibitors (TKI) as first-line treatments. However, when progression occurs, the therapeutic options are limited. Understanding crucial biological pathways could lead to a greater understanding of the natural history of the disease, which could help to overcome the mechanism of resistance and to develop new treatments. The clinical significance of homologous recombination deficiency (HRD) in RCC remains to be investigated. To improve the knowledge about this topic, we conducted a narrative review to summarize the current evidence on HRD-related variations and signatures in RCC, together with their prognostic and predictive implications. Preliminary evidence indicates that canonical HRD variants (BRCA1/2) are infrequent in RCC, while broader DNA damage response (DDR) alterations like BAP1, PBRM1, ATM, and SETD2 are more prevalent. Elevated HRD genomic scores in clear-cell RCC correlate with a worse prognosis and an immunologically exhausted microenvironment. From a therapeutic point of view, PARP inhibitor monotherapy has exhibited initial efficacy in small cohorts with high levels of DDR mutation, yet remains investigational for RCC.

## Simple Summary

To date, the role of HRD in RCC remains unclear. Non-canonical HRD variants like BPA1, PBRM1, ATM, and SETD2 are more frequently observed than canonical ones. Non-canonical HRD variants could be prognostic and predictive for the activity of ICI and PARPinhibitors. Therapeutic options like ATR inhibitors should be investigated for the treatment of HRD RCC.

## 1. Introduction

Renal cell carcinoma (RCC) is a common cancer, with an estimated incidence of 81 800 new cases in 2023, making it the sixth- and ninth-most common cancer in males and females, respectively [1,2].

According to the WHO 2022 classification, RCC includes more than 20 subtypes of malignant tumors, such as clear-cell carcinoma (ccRCC), which constitutes over 80% of RCC cases, as well as non-clear-cell subtypes [3,4].

Although renal cell carcinoma (RCC) can be diagnosed early and effectively managed through surgical or ablative methods, approximately one-third of cases may exhibit or develop metastases, and 20–40% may experience recurrence following nephrectomy for localized disease [5].

Recently, the KEYNOTE-564 phase III trial demonstrated the advantages of adjuvant pembrolizumab in patients with resected clear-cell RCC at intermediate–high or high risk of recurrence, including those with resected stage M1 disease. Despite these significant achievements, approximately 40% of patients develop a relapse within five years [6,7].

Until now, the standard treatment for metastatic RCC has included dual immune-checkpoint inhibitors (ICIs) Nivolumab and Ipilimumab, or tyrosine kinase inhibitors (TKIs) and/or mammalian target of rapamycin inhibitors (mTORi), either alone or together with an ICI. Patients exhibiting disease progression following first-line therapy with a TKI combined with an ICI or dual ICI therapy may consider treatment alternatives that include previously unutilized TKIs such as cabozantinib, lenvatinib in conjunction with everolimus, or the hypoxia-inducible factor-2 alpha (HIF-2α) inhibitor, belzutifan [8,9,10,11].

Most patients with RCC have a deletion of chromosome 3p and genomic alterations in the Von Hippel–Lindau (VHL) tumor suppressor allele, accompanied by the subsequent loss of other tumor suppressor genes, including PBRM1, SETD2, BAP1, and/or KDM5C. These genes contribute further to genomic instability [12].

Genomic instability and deficiencies in DNA repair, especially in homologous recombination repair (HRR), promote carcinogenesis and determine treatment sensitivity in several malignancies [13].

In recent years, homologous recombination deficiency (HRD) has emerged as a significant predictive biomarker in different cancer types. While the presence of HRD in adrenal cortical carcinoma indicates a poorer clinical prognosis relative to non-HRD patients, HRD-positive ovarian cancer patients can benefit from PARP inhibitor therapy [14,15,16,17].

In RCC, the landscape and clinical relevance of HRD are as yet inadequately explored [18].

HRD may also increase the immunogenicity of tumor cells, allowing them to benefit from immunotherapy, and it may be linked to poorer prognosis [13]. Analyzing HRD in RCC could help to better predict outcomes, guide targeted therapies and immunotherapies, and facilitate early screening of high-risk populations, which is of great importance for improving clinical practice

The aim of this study is to revise the existing knowledge on the clinical significance of HRD variations and signatures in RCC, encompassing their prognostic and predictive value to provide a rationale for the use of drugs already in use for other conditions or for the development of new drugs.

## 2. Materials and Methods

From 2000 to 2025, a study search was performed on MEDLINE (Ovid), Embase (Ovid), Web of Science Core Collection, Scopus, Cochrane Central, ClinicalTrials.gov, and WHO ICTRP. Gray literature and conference abstracts from ASCO, ESMO, AACR, and IKCS were reviewed. The terms used for the literature research are described in the Supplemental Material. For the keyword selection, we started with the study topic, deconstructing it into essential concepts and subsequently employing synonyms, related terminology, and controlled vocabularies such as MeSH to identify a broad array of terms. Additionally, we selected relevant trials from the ClinicalTrials.gov database (https://www.clinicaltrials.gov/ accessed on 20 October 2025). Two reviewers screened titles, abstracts, and then full texts, prioritizing peer-reviewed publications above preprints or non-academic sources, with a focus on well-structured research featuring robust data and transparent procedures, and according to the journal’s reputation.

The reference lists of included studies were screened.

The studies included in this review met the following criteria:

1. Published in English;
2. Conducted both in vitro for preclinical evidence and in human subjects for clinical trials;
3. Evaluated the role of HRD in solid tumors with a particular focus on RCC;
4. Clinical trials, cohort studies, comparative studies, or reviews specifically addressing the activity of drugs involved in the DNA repair system dedicated to, and performed with, a cohort of RCC patients.

The exclusion criteria included the following:

1. Articles that were not published in English;
2. Focused on hematological diseases;
3. Case reports/commentaries/editorials/opinion pieces without original data included;
4. HRD variants of uncertain significance were excluded.

The identification and selection process adhered to SANRA principles, ensuring transparency and reproducibility [19].

## 3. Results

### 3.1. DNA Damage Response

**Figure 1:**
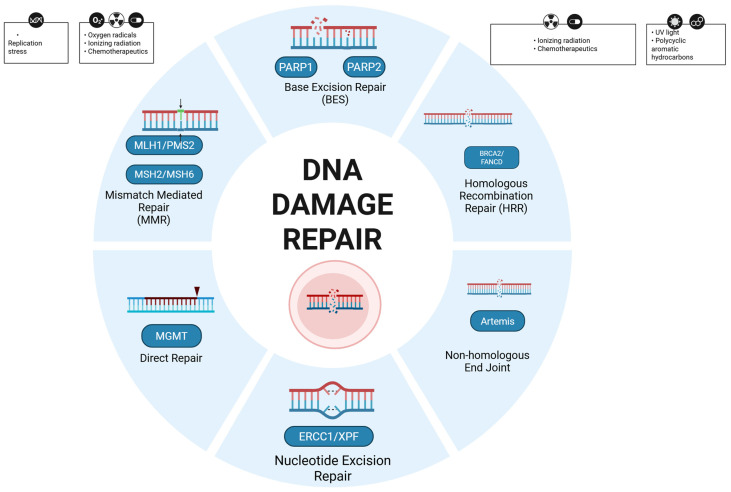
Mechanisms of DNA damage repair. Created in BioRender. B., A. (2025) https://BioRender.com/t6bpq1u (accessed on 20 October 2025).

The DNA damage response (DDR) comprises a network of different repair pathways that address distinct classes of genomic lesions, most notably single-strand breaks (SSBs) and double-strand breaks (DSBs) (Figure 1) [20]. SSBs are the most frequent form of DNA damage. As demonstrated in several in vitro experiments on human tumor cell cultures, within BER, PARP1/2 function as central players in SSBs and catalyze poly (ADP-ribose) (PAR) synthesis on chromatin-associated proteins, thereby recruiting downstream factors that coordinate end processing and ligation [21,22,23,24].

DSBs are mostly repaired by two mechanisms: non-homologous end joining (NHEJ) and homologous recombination repair (HRR). Non-homologous end joining (NHEJ) is predominant in G1, while homologous recombination repair (HRR) is preferred in S/G2 [20,25,26].

HRR is initiated by the MRN complex (MRE11–RAD50–NBS1), which recognizes DSBs and coordinates the recruitment of ATM and BRCA1/2 [27].

Downstream, CHEK1/2, PALB2, and p53 enforce checkpoint activation and facilitate the loading of RAD51 onto resected DNA ends, enabling strand invasion, D-loop formation, and high-fidelity DNA synthesis using the sister chromatid as a template [28].

**Figure 2:**
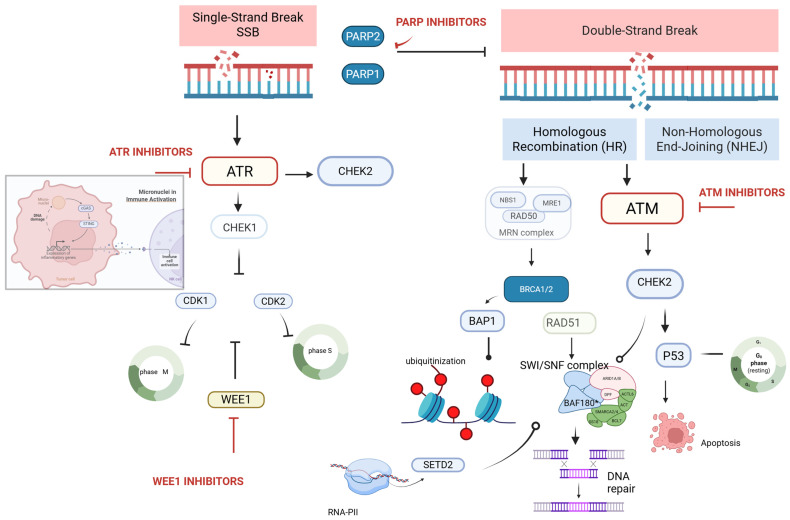
Schematic representation of the DDR pathway and investigational agents used in RCC. PARP 1 and PARP 2 prevent the conversion of SSBs to double-strand breaks, DSBs. PARP inhibitors increase the DSB in tumoral cells, which is the rationale for their use in HRD RCC. SSB leads to the activation of ATR and CHEK1. The latter promotes the inhibition of CDK2 and CDK1. WEE1 regulates the CDKs’ activity in a negative manner, playing an essential role in controlling S and M phase entry. The MRN complex recognizes DSBs and activates ATM, which plays a major role in DSB repair. Blocking both PARP and ATM prevents DNA repair activities. SETD2, activated by RNA polymerase II, can activate the SWI/SNF complex, which can lead to DNA repair. In patients with SETD2 loss, the use of ATR inhibitors could increase cytosolic DNA with the activation of the GAS-STING pathway and improve the immunogenicity of the tumor. Finally, POLθ is involved in NHEJ, and its inhibition could lead to increased lethality in HRD RCC. Created in BioRender. B., A. (2025) https://BioRender.com/iuejvuo (accessed on 20 October 2025).

These processes have been summarized in Figure 2.

Under physiological conditions, PARP1-mediated repair of SSBs prevents their conversion into replication-associated DSBs. PARP inhibitors (PARPi) impede this process, increasing DSB burden and rendering cells that are defective in HRR, classically those harboring BRCA1/2 alterations, highly vulnerable. The consequent selective cell death of HRR-deficient tumor cells upon PARP inhibition constitutes synthetic lethality [29,30].

In addition to classical BRCA1/2 alterations, several additional mutations have been detected, including in genes associated with chromatin remodeling, which are located at 3p21, such as polybroma 1 (PBRM1, ~40%), SET domain-containing 2 (SETD2, ~10%), and BRCA1-associated protein 1 (BAP1, ~12–14%) [18,31].

### 3.2. BAP1 (BRCA1-Associated Protein 1)

BAP1 (BRCA1-associated protein 1) is a tumor suppressor gene localized on chromosome 3p21.1 that encodes a 90-kDA protein localized to the nucleus. BAP1 serves as a crucial tumor suppressor in ccRCC, functioning through chromatin control and the DNA damage response, particularly in homologous recombination repair [32]. The inactivation of BAP1 leads to poor cell cycle regulation and cell proliferation, resulting in tumorigenesis, characterizing a clinically unique subtype of ccRCC, marked by aggressive characteristics and an inflammatory but immunosuppressed tumor microenvironment [33,34]. Somatic BAP1 mutations or deletions are seen in around 5–15% of cases of ccRCC and are far less prevalent in non-clear cell histologies. BAP1 is predominantly mutually exclusive with PBRM1 at the clonal level. When co-inactivated, these tumors exhibit notably detrimental histopathologic characteristics. In the genomic analysis of the primary tumor tissue of the TRACERx Renal study, the multi-region sequencing underscores intratumoral heterogeneity and validates clonal mutual exclusivity patterns associated with BAP1 and PBRM1/SETD2 [35,36].

In a retrospective analysis on primary tissue, BAP1-deficient RCCs exhibited a higher prevalence of elevated WHO/ISUP grades and unfavorable characteristics in comparison to PBRM1 mutant tumors [37,38].

The absence of nuclear BAP1 detected by immunohistochemistry (IHC) serves as an effective surrogate for inactivating mutations and is associated with clinical outcomes; localized or segmental loss may indicate subclonality [36,39]. BAP1 mutation or deletion is associated with markedly poorer survival in ccRCC, irrespective of stage and other confounding factors. A pivotal translational study on both tissue and blood samples revealed that BAP1 mutant tumors exhibited significantly reduced overall survival compared to PBRM1 mutant tumors, with a median OS of 4.6 years in one against 10.6 years in the other [40,41].

Germline pathogenic mutations in BAP1 result in an autosomal dominant tumor predisposition syndrome, increasing the risk for uveal melanoma, mesothelioma, and renal cell carcinoma—predominantly clear cell—and at a younger median age compared to sporadic occurrences. The incidence of renal cell carcinoma (RCC) in families with BAP1 tumor predisposition syndrome (TPDS) is significantly increased; genetic counseling and customized monitoring are advised for suspicious families [37,38,39,40,41].

The loss of BAP1 is associated with homologous recombination biology, functioning in conjunction with RAD51 family proteins, and has been suggested as a surrogate for homologous recombination deficiency in certain fractions of ccRCC [42].

In ccRCC, patients exhibiting low or absent BAP1 expression demonstrated overexpression of EZH2, which correlated with reduced overall survival and unfavorable prognosis. Inhibition of EZH2 appears to diminish tumor proliferation and metastasis in both in vitro and in vivo models. However, these recommendations were undermined by the unfavorable outcomes of clinical trials investigating EZH2 inhibitors [43].

Initial clinical data investigating PARP inhibition encompass BAP1/DDR-changed tumors and indicate a possible efficacy of PARP inhibitors as a treatment option. Interim analysis data from the phase 2 ORCHID experiment (ClinicalTrials.gov. NCT03786796) demonstrated that single-agent olaparib elicited responses in patients with renal cell carcinoma (RCC) harboring BAP1 or other DNA repair (DDR) gene mutations. Olaparib produced a disease control rate (DCR) of 18% in this group (*n* = 11). The objective response rate (ORR) was 9% and 27% of patients had a reduction in tumor size, including those with BAP1-mutated disease. One patient obtained a sustained partial response (PR) to treatment, while the other exhibited extended SD for a duration of 10 months [44,45]. Nonetheless, the evidence is still exploratory.

### 3.3. RAD51

RAD51 is a crucial protein in the homologous recombination DNA repair pathway and may serve as a marker for HR functionality. Significant evidence demonstrates that RAD51 is absent in defective homologous recombination pathways [46,47,48]. In retrospective studies performed on tissue samples from patient cohorts, RAD51 was shown to be a positive predictive biomarker of treatment response across multiple cancers, such as platinum-based chemotherapy in breast cancer and esophageal squamous cell carcinoma, as well as PARPi in pancreatic and prostate cancers [48,49,50]. However, limited investigations have converged on the correlation between HRD-associated RAD51 and treatment responses in ccRCC [51].

In ccRCC, the dysregulation of the RAD51 pathway may result in characteristics of a homologous recombination deficit. In fact, PBRM1 and RAD51 expressions are linked to traits akin to homologous recombination deficit and a possible association with efficacy from immune checkpoint inhibitors. The absence of RAD511 resulted in a more pronounced advantage from immunotherapy compared to Everolimus. RAD51 deficiency patients demonstrated a survival benefit from immunotherapy (Hazard Ratio [HR]: 0.498, 95% Confidence Interval [CI] 0.322–0.771, log-rank *p*  <  0.001), whereas no benefits were observed from Everolimus (HR: 0.946, 95% CI 0.583–1.534, log-rank *p*  =  0.706). In a cohort of 62 patients undergoing immunotherapy, those with RAD51 deletion demonstrated significant survival benefits (HR: 0.076, 95% CI 0.015–0.378, log-rank *p*  <  0.001). In contrast, among the sample of 66 patients undergoing TKI monotherapy, no significant differences were noted (HR: 1.056, 95% CI 0.375–2.974, *p* = 0.906) [52]. These data, however, are preliminary and need further confirmation.

Recent observations on in vitro and in vivo preclinical studies on cell cultures and patient-derived xenografts (PDx) indicate that PARP inhibitors are effective in treating prostate and breast cancers, which are characterized by the loss of RAD51 foci [53].

### 3.4. PBRM1

PBRM1 encodes BAF180, a subunit of the SWI/SNF chromatin-remodeling complex. PBRM1 is mutated in about 40% of all ccRCC cases and is the second most identified mutation in ccRCC [54,55,56]. Previous published studies on patient tumor samples have reported that PBRM1 mutations, usually truncating ones, lead to a loss of PBRM1 function. All these mutations were identified within the framework of the loss of heterozygosity on chromosome 3p, specifically involving allelic loss at 3p21 [57,58,59,60,61,62]. In renal cell carcinoma, tumors with PBRM1 deletion exhibit increased replication stress, DNA double-strand breaks, and genomic instability [63].

PBRM1 deletion is frequently associated with lower-grade RCC compared to BAP1 mutations, while the results correlate differently among studies.

Initial findings have indicated a heightened benefit of ICI in PBRM1 mutant ccRCC. In particular, Braun et al. confirmed the association between PBRM1 alterations and the response to Nivolumab in the tissue samples of a validation cohort of patients with metastatic clear cell renal cell carcinoma enrolled in a prospective clinical trial and treated with Nivolumab or Everolimus [63].

PBRM1-mutated patients showed increased PFS (HR, 0.67; 95% CI, 0.47–0.96; *p*  =  0.03) and OS (HR, 0.65; 95% CI, 0.44–0.96; *p*  =  0.03), while among patients treated with everolimus, there was no evidence of correlation to treatment response.

Additional data from the RECORD-3 research indicated that individuals with PBRM1 mutant tumors exhibited favorable responses to antiangiogenic treatment. Results from the IM-motion 150 research similarly indicated that patients with PBRM1 mutant tumors exhibited superior responses to sunitinib compared to those with wild-type tumors [64,65].

Despite these findings, to date, the predictive value of PBMR1 is still controversial, which is likely due, in part, to the fact that PBRM1 alone lacks the capacity to define a homogenous molecular ccRCC subtype [61].

However, further investigations present inconsistent or diminished benefits, lacking validation for clinical selection. In non-clear cell renal cell carcinoma (nccRCC), PBRM1 mutations are infrequent and lack a confirmed prognostic or predictive significance.

### 3.5. SETD2 (Homologous Recombination/Chromatin)

SETD2 encodes the sole human histone H3 lysine-36 trimethyltransferase (H3K36me3) and resides on chromosome 3p21.31—a region frequently deleted in ccRCC. In large ccRCC cohorts, SETD2 is among the most recurrently altered tumor-suppressor genes (≈10–15% in primaries) and appears further enriched in metastatic disease (≈30%), underscoring its role in progression [66].

Mechanistically, SETD2-dependent H3K36me3 links transcriptionally active chromatin to high-fidelity DNA double-strand break (DSB) repair by homologous recombination (HR). The H3K36me3 mark is read by the PWWP domain protein LEDGF/PSIP1, which helps recruit CtIP and downstream HR factors to promote DNA end resection and RAD51 loading; genetic or pharmacologic abrogation of SETD2 diminishes H3K36me3 and compromises HR efficiency, increasing genomic instability [67].

Beyond HR, H3K36me3 provides an epigenetic landing pad for the mismatch repair (MMR) machinery: the MSH6 PWWP domain binds H3K36me3 to recruit MutSα to replicate chromatin. Loss of SETD2, therefore, weakens MMR surveillance, contributing to mutational burden even without classical microsatellite instability—an effect particularly relevant in ccRCC, where 3p loss and SETD2 mutation co-occur [68].

The consequences for chromatin of SETD2 loss extend to nucleosome reassembly behind replication forks and RNA processing within transcribed gene bodies. In ccRCC models, biallelic SETD2 inactivation produces replication stress, elevated DNA damage in vivo, and a pattern of branched tumor evolution, implicating SETD2 in preserving chromosomal integrity across the S phase [69].

SETD2-deficient RCC cells display heightened dependence on replication-stress response pathways; preclinical work shows synergy between SETD2 loss and ATR inhibition, with augmented cGAS–STING activation and antitumor immunity. This is the rationale for clinical exploration of ATR-pathway inhibitors (alone or in combinations) in SETD2 mutant tumors [70,71].

In ccRCC, SETD2 loss (or loss of its mark, H3K36me3) is associated with a poor outcome. In a large Mayo cohort, the loss of H3K36me3 by IHC—a functional readout of SETD2—was linked to ~two-fold higher risks of RCC-specific death and progression and remained significant after adjustment in risk-stratified models; by contrast, SETD2 DNA/mRNA alone was less consistently prognostic [69,70,71]. Experimental clinical and genomic studies further show that SETD2 loss promotes metastasis and creates actionable chromatin dependencies, reinforcing its adverse biological behavior.

Across datasets, the functional deficit (H3K36me3 loss) outperforms genotype alone for outcome discrimination, while reduced SETD2 expression similarly tracks with unfavorable survival.

A recent multi-gene 3p analysis conducted in a preclinical study on cell line and cell culture in mice suggests a possible negative predictive effect of SETD2 alterations for ICI-based regimens, whereas pan-cancer work links SETD2 mutation to a more “inflamed” phenotype.

RCC studies indicate that SETD2-deficient tumors can be immune-cold and ICI-resistant unless innate sensing is pharmacologically engaged. By contrast, preclinical RCC models suggest that ATR pathway inhibition shows synthetic vulnerability to ICI treatment in SETD2-loss with cGAS–STING activation [70].

### 3.6. CHEK2

In ccRCC, CHEK2 is disproportionately prevalent among germline variations compared to the general population; nevertheless, its prognostic significance within ccRCC is yet to be elucidated [72].

CHEK2 encodes a checkpoint kinase in the DNA-damage response (the ATM–CHEK2–p53 axis) that phosphorylates targets such as p53, BRCA1, and CDC25 to enforce cell-cycle arrest, repair, or apoptosis; disruption of this pathway can promote genomic instability in renal tumors [73]. From a hereditary standpoint, pathogenic CHEK2 variants—well established in other cancers like breast—appear to confer at most a low-to-moderate increase in RCC risk: associations (including with I157T and c.1100delC) have been reported but are inconsistent and show limited penetrance [74].

Clinical cohorts have also noted CHEK2 variants in some non-clear-cell histologies, often without a distinctive phenotype, suggesting they may be incidental (“passenger”) findings on multigene panels [75].

There is no established predictive role for immune checkpoint inhibitors or PARP inhibitors in renal cell carcinoma. Preclinical data suggest CHEK2 deficiency may enhance PD-1/PD-L1 response via immune microenvironment effects and higher neoantigen load, but this is not yet confirmed prospectively in RCC [72].

### 3.7. ATM

In ccRCC, abnormalities in ATM are rare. ATM plays a pivotal role in DSB repair as part of the HR process. In 2019, Ren et coll explored the relationship between the expression of ATM and ccRCC. In a cohort of 110 patients, the ATM low-expression group had a significantly shorter survival than those with high ATM expression. A validation study is needed to confirm this trend. Although ATM deletion may increase sensitivity to ATR inhibitors in other tumors, there are no data on RCC, and the benefit of PARP inhibitors has yet to be proven [76].

Preclinical evidence suggests that pharmacological inhibition of the DNA damage-sensing kinase ataxia telangiectasia and Rad3-related protein (ATR) with selective drugs like gartisertib could induce antiproliferative effects in ccRCC cells [77]. The development of Gartisertib was stopped to prioritize the clinical evaluation of M1774, a potent and highly selective inhibitor of ATR protein kinase, both as a monotherapy and in combination with niraparib in patients with advanced solid tumors (NCT04170153) [78]. Another ATR inhibitor, Ceralasertib, is being evaluated alone or in combination with olaparib or durvalumab in a phase II study enrolling patients with RCC and other solid tumors (ClinicalTrials.gov ID NCT03682289)

### 3.8. BRCA1 and BRCA2

RCC presents a lower prevalence of BRCA1/2 mutations than breast or ovarian cancer. Pathogenic mutations in ccRCC are few and are not more prevalent compared to other established ccRCC drivers [79]. No reliable predictive effect has been established, and there is no randomized data related to RCC demonstrating the efficacy of treatment based on BRCA status [80]. Also, for nccRCC, data is scarce. BRCA1/2 mutations seem infrequent; no prognostic or predictive significance has been established [79,80].

### 3.9. PALB2

In ccRCC, PALB2 pathogenic mutations are infrequent to rare in tumor or germline assessments; the existing literature does not endorse a consistent prognostic significance.

There is no RCC-specific evidence indicating that PALB2 status predicts the response to PARP inhibitors. Their application is limited to clinical studies. In nccRCC, data are exceedingly scarce, and no validated implications exist [81,82,83].

### 3.10. HRD Scores/Signatures in RCC

In renal cell carcinoma (RCC), homologous recombination deficiency (HRD) has been assessed largely through genomic scar metrics and mutational signature classifiers adapted from ovarian and breast cancers, but RCC-specific validation remains limited. Scar-based approaches quantify the footprints of defective HR repair—most commonly the composite of the loss of heterozygosity (LOH), telomeric allelic imbalance (TAI), and large-scale state transitions (LST), or related genome instability scores [84]. Signature-based methods include HRDetect (a WGS-trained classifier integrating SBS3, indel signatures enriched for micro-homology, and HR-linked rearrangements) and SigMA (which infers SBS3 from exome/panel data) [85,86,87]. In ccRCC, HRD-high by these methods appears uncommon and cutoff-dependent; nevertheless, higher HRD scores have been correlated with worse survival and with an exhausted T-cell microenvironment in retrospective cohorts [88]. Interpretation is complicated by RCC’s genomic context and due to its high intratumoral heterogeneity, all of which could affect reproducibility across regions and time [35,89].

As predictive biomarkers, RCC HRD scores remain unproven. Platinum chemotherapy is not standard in RCC, and PARP inhibitor (PARPi) monotherapy/combination signals derive from small DDR-altered series without RCC-specific, threshold-anchored selection. A practical way forward is to pair genomic scars with functional HRD readouts—notably, RAD51 foci assays calibrated for FFPE—as well as activity-based functional tests that capture live HR proficiency across tumor types [90,91,92]. In parallel, curated DDR gene panels (BRCA1/2, PALB2, ATM/CHEK2, RAD51 family) augmented by RCC-salient chromatin genes (SETD2, BAP1, PBRM1) may increase biological specificity. PARPi exploration in RCC illustrates the field’s investigational status and the need for biomarker-driven prospective trials [44].

### 3.11. HRD Variants and Therapeutic Options

Poly(ADP-ribose) polymerase (PARP) inhibition in renal cell carcinoma (RCC) is biologically plausible via synthetic lethality in tumors with impaired DNA-damage repair (DDR), notably BAP1 and other DDR lesions, yet clinical translation in metastatic RCC (mRCC) remains preliminary [6].

As reported previously, the clearest prospective signal so far is from the single-arm, phase II ORCHID study of olaparib monotherapy in molecularly selected mRCC, which has been reported to have early antitumor activity, particularly in BAP1-mutated disease, prompting ongoing expansion; however, a randomized evidence base is absent [44]. Combination strategies are being tested to potentiate their efficacy, including niraparib plus cabozantinib (VEGF/MET/AXL TKI) and talazoparib plus avelumab (PD-L1), the latter showing no objective responses and short median PFS across genomically defined cohorts, underscoring the need for sharper biomarker selection and optimized partners [93,94]. A niche but important avenue targets hereditary fumarate hydratase-deficient RCC (HLRCC) with pamiparib + temozolomide [95]. Overall, PARP inhibitors in mRCC are investigational; outside of trials, they should not guide standard therapy at present (see Table 1).

Beyond PARP inhibitors, ATR inhibition is the most active area. A phase I/II program tests ceralasertib alone or with durvalumab or olaparib and list RCC among eligible solid tumors, though no RCC-specific, positive read-out has been reported yet [96]. WEE1 inhibition (adavosertib/AZD1775) has been prospectively evaluated in SETD2-altered tumors—including clear-cell RCC—with no objective responses but some prolonged disease control, arguing against monotherapy in this biomarker subset [97].

DNA-PK inhibitors (CC-115, peposertib/M3814) remain investigational. The CC-115 shows preclinical RCC activity via DNA-PK/mTOR blockade and has phase I safety data in mixed solid tumors, while peposertib is being explored preclinically and in early trials (including combinations with ATR inhibitors), with an RCC-relevant rationale but no clinical efficacy signal in RCC yet [98]. Finally, POLθ (DNA polymerase-theta) inhibitors (e.g., novobiocin) are in first-in-human studies for DDR-altered cancers and could be biologically attractive for HRD-like RCC subsets, but no results in RCC are available [99] (Table 2).

## 4. Discussion

Several genes are involved in DDR pathways in order to maintain genomic stability. Disruption in DDR pathways can facilitate cancer growth, justifying the exploitation of repair defects for therapeutic interventions in several cancers [100].

Immunogenic tumor types such as melanoma and mesothelioma could provide a useful blueprint for integrating HRD biology with immune-based therapies in RCC. In melanoma, early studies combining PARP inhibitors with immunotherapy could help to define combination strategies. Similarly, Mesothelioma, which, like RCC, is frequently characterized by BAP1 loss, has become a testing ground for the therapeutic implications of PARP inhibitors [33,101,102].

Current research indicates that HRD in RCC holds biological significance in subgroups characterized by DNA damage response (DDR) and epigenetic abnormalities [103].

HRD may have a negative prognostic impact in RCC, although its use as a predictive biomarker for PARP inhibitors or other treatments is still under investigation [104].

Further validation is needed to confirm DDR pathway alterations as a predictive marker of immunotherapy response in RCC and to select patients who may be responsive to a potential PARPi combination therapy with immunotherapy [44,93,94,95,96,97,104]. This challenge is consistent with broader immuno-oncology observations, where intrinsic and acquired resistance to PD-1–targeted therapies, driven by impaired antigen presentation, immunosuppressive tumor microenvironments, and altered tumor-infiltrating lymphocytes, continues to limit the therapeutic efficacy of checkpoint blockade across multiple cancers [105].

Understanding how DDR pathway alterations could remodel the tumor microenvironment will provide future opportunities to treat patients with RCC cancer who harbor DDR-pathway alterations [80,106].

To summarize the data presented, non-canonical HRD alterations seem to play an interesting role in RCC. BAP1 loss in ccRCC defines an aggressive, HRD-like subset, in which early clinical data with the PARP inhibitor olaparib have shown signals of activity, including tumor shrinkage and durable disease control in patients with BAP1/DDR alterations. RAD51 deficiency similarly marks an HRD-like phenotype and is associated with a pronounced survival benefit from immune checkpoint inhibitors (but not from everolimus or TKIs), supporting its potential as a predictive biomarker for immunotherapy and PARP inhibition. PBRM1, one of the most frequently mutated genes in ccRCC, has been linked in several studies to improved outcomes with nivolumab and with antiangiogenic agents such as sunitinib, suggesting that PBRM1 status may help refine treatment selection despite some remaining controversy [18,19,20,21,22,23,24,25,26,27,28,29,30,31,44,61].

To date, no PARP inhibitor has been demonstrated as an effective treatment in RCC, and specifically, no phase III results have yet been published. A better biological selection of patients enrolled in an interventional clinical trial could be useful.

Preclinical data suggest that SETD2 deficiency increases replication stress and may sensitize tumors to ATR inhibition, providing a strong rationale to explore ATR inhibitors in combination with immunotherapy in this molecular subset. Future studies should therefore prioritize rational combination regimens to translate these insights into more precise treatment options for RCC patients [70,71].

Another point of discussion is on the methodology used to identify these alterations. The HRD phenotype is dynamic and subject to change over time, and the data obtained from tissue samples may not necessarily be indicative of the HRD status at the time of treatment. To overcome these limitations, functional tests and a liquid biopsy approach for HRD testing could be more sensitive in capturing the tumor heterogeneity, but need further refinement before being implemented due to the risk of false negative or positive results [18].

Finally, most HRD metrics have been directly borrowed from other cancers, and this creates several limitations. These assays and cut-offs were calibrated in tumors with high rates of BRCA1/2-type HRD and may lack sensitivity and specificity in RCC, where alternative HRR gene alterations are more frequent [107].

This review is subject to several limitations. First, it is not a systematic review, and the quality of the included literature was not formally evaluated. To overcome this limitation, guided by the research question, we followed a clear and systematic approach to the literature search to ensure reproducibility, and then synthesized the results by comparing the studies to identify themes and gaps. Furthermore, we tried to summarize and highlight the role of HRD in RCC that could lead to improving the therapeutic outcomes of this disease.

Future trials should stratify patients according to histology, utilizing standardized HRD assays and RCC-specific thresholds or incorporating genomic scars, functional homologous recombination deficiency readouts, and transcriptome markers.

The importance of molecular data in precision cancer therapy is increasingly recognized, as is the integration of artificial intelligence for data analysis, but a challenge remains in improving the criteria, costs, and timing to improve access to these important diagnostic tools for patients outside of clinical trials [108].

## 5. Conclusions

HRD in RCC is challenging. Early evidence on its prognostic significance is growing, while its predictive value is still ambiguous. Future trials using standardized HRD tests and histology-specific criteria are needed.

## Figures and Tables

**Table 1:** Published trials on the use of PARP- inhibitors in mRCC.

Trial	Phase	Population (Biomarker/Histology)	Treatment	Status/Key Read-Outs	NCT
ORCHID	II (single-arm)	mRCC with DDR mutations (e.g., BAP1, BRCA1/2, ATM, etc.)	Olaparib monotherapy	Recruiting with interim activity: responses seen, including in BAP1-mutated RCC; expansion ongoing.	NCT03786796
NCT04068831	II (single-arm)	mRCC, VHL-def ccRCC; FH/SDH-def RCC; renal medullary carcinoma	Talazoparib + Avelumab	Negative: no objective responses; median PFS 3.5 mo (cohort 1) and 1.2 mo (cohort 2); safety acceptable.	NCT04068831
NICARAGUA	I/II	Advanced urothelial and RCC (mixed cohorts)	Niraparib + Cabozantinib	Phase I established dosing with preliminary activity; phase II emphasized urothelial cancer; RCC-specific efficacy not yet confirmed.	NCT03425201
NCT04603365	II	Hereditary leiomyomatosis and RCC (can present as mRCC)	Pamiparib + Temozolomide	Ongoing; evaluating response rate and PFS in FH-deficient RCC.	NCT04603365

**Table 2:** Summary of the most interesting studies on agents targeting HRD RCC other than PARP- Inhibitors.

Target	Agent	Administration Modality	Study Design	Status/Notes
ATR	Ceralasertib (AZD6738)	Monotherapy; +durvalumab; +olaparib	Phase I/II solid tumor studies that include RCC cohorts/eligibility.	Ongoing/active; early signal mainly outside RCC so far.
WEE1	Adavosertib (AZD1775)	Monotherapy in SETD2-altered tumors (incl. ccRCC)	Phase II: no ORR in SETD2-altered ccRCC; some durable DCR.	Negative for RCC as monotherapy; combinations under exploration.
DNA-PK	CC-115 (dual DNA-PK/mTOR inhibitor)	Monotherapy	Phase I safety in mixed solid tumors (not RCC-specific).	Investigational; no RCC efficacy data in patients.
DNA-PK	Peposertib (M3814)	With radiation/with ATR inhibitor (tuvusertib/M1774)	Ongoing early-phase combo trials in solid tumors.	Very early; no RCC clinical outcomes yet.
POLθ	Novobiocin	Monotherapy (DDR-altered cancers)	First-in-human phase I; histology-agnostic.	Dose-finding/biomarker-driven; efficacy unknown in RCC.

## Data Availability

The original contributions presented in this study are included in the article. Further inquiries can be directed to the corresponding author.

## Supplementary Materials

The following supporting information can be downloaded at: https://www.mdpi.com/article/10.3390/curroncol32120690/s1, Table S1: The table summarizes HRD alterations in RCC.

## Abbreviations

The following abbreviations are used in this manuscript:

ccRCC	Clear Cell Renal Cell Carcinoma
DDR	DNA damage repair
DSB	Double-strand break
ICI	Immune checkpoint inhibitors
NHEJ	Non homologous End-joining
nccRCC	Non clear cell Renal Cell carcinoma
PARPi	Poly (ADP-ribose) polymerase inhibitors
PDx	patient-derived xenograft
RCC	Renal Cell Carcinoma
SSB	Single Strand Break
TKI	Tyrosine Kinase Inhibitors
