# Supplementary material for: The Role of Homologous Recombination Deficiency (HRD) in Renal Cell Carcinoma (RCC): Biology, Biomarkers, and Therapeutic Opportunities
Source: Curr Oncol. 2025 Dec 7;32(12):690. doi: 10.3390/curroncol32120690 (PMC12731853; doi:10.3390/curroncol32120690)
Supplement: Supplementary file 1 [file curroncol-32-00690-s001.zip › curroncol-3977821-supplementary.pdf]

The following keywords have been used:"Kidney Neoplasms"[Mesh] OR renal cell carcinoma[tiab] OR RCC[tiab] OR "renal carcinoma"[tiab] OR "clear cell renal"[tiab] OR papillary[tiab] OR chromophobe[tiab])AND ("Homologous Recombination"[Mesh] OR "homologous recombination deficiency"[tiab] OR HRD[tiab] OR "DNA repair defect*"[tiab] OR "DNA damage repair"[tiab] OR HRR[tiab] OR BRCA1[tiab] OR BRCA2[tiab] OR PALB2[tiab] OR ATM[tiab] OR CHEK2[tiab] OR CHEK1[tiab] OR RAD51*[tiab] OR BAP1[tiab] OR PBRM1[tiab] OR SETD2[tiab] OR VHL[tiab] OR "genomic scar"[tiab] OR "HRD score"[tiab] OR HRDetect[tiab] OR "loss of heterozygosity"[tiab].

Supplementary Table S1. The table summarizes HRD alterations in RCC.

Specific category	Markers
***Canonical HRD**	BRCA1, BRCA2, PALB2, RAD51 pathway genes
***Non-canonical HRD**	BAP1, PBRM1, SETD2 (mutations/LOF, epigenetic changes)
**Genomic scar HRD scores**	Composite HRD score, LOH, TAI, LST
**Functional HRD assays**	RAD51 foci assay, ex vivo DSB repair tests

*HRD (canonical/non-canonical), HRD score, functional assay could have a predictive value in response to platinum based compounds or PARPi while BAP1, PBRM1, SETD2 alteration seem have a prognostic significance.
